# Rapidly Expanding Range of Highly Pathogenic Avian Influenza Viruses

**DOI:** 10.3201/eid2107.150403

**Published:** 2015-07

**Authors:** Jeffrey S. Hall, Robert J. Dusek, Erica Spackman

**Affiliations:** US Geological Survey National Wildlife Health Center, Madison, Wisconsin, USA (J.S. Hall, R.J. Dusek);; US Department of Agriculture, Athens, Georgia, USA (E. Spackman)

**Keywords:** HPAIV, highly pathogenic avian influenza virus, reassortment, wild birds, movement, viruses, influenza, bird migration

## Abstract

The movement of highly pathogenic avian influenza (H5N8) virus across Eurasia and into North America and the virus’ propensity to reassort with co-circulating low pathogenicity viruses raise concerns among poultry producers, wildlife biologists, aviculturists, and public health personnel worldwide. Surveillance, modeling, and experimental research will provide the knowledge required for intelligent policy and management decisions.

The recent introduction of highly pathogenic avian influenza (HPAI) subtype H5N8 virus into Europe and North America poses major risks to poultry industries, zoologic collections, and wildlife populations; thus, this introduction warrants continued and heightened vigilance. First discovered in early 2014 in poultry and wild birds in South Korea, HPAI H5N8 virus apparently arose in China from reassortment events between HPAI subtype H5N1 virus (clade 2.3.4.4) and several low pathogenicity viruses (LPAIVs) (*1*–*3*). The H5N8 virus was subsequently detected in waterfowl in Russia in September 2014, and since then, H5N8 virus and reassortants have been detected in poultry and wild birds in Europe (Netherlands, Germany, Italy, the United Kingdom, Hungary, and Sweden), Taiwan, Japan, Canada (British Columbia), and the western and central United States (Washington, Oregon, California, Idaho, Utah, Minnesota, Missouri, Arkansas, Kansas, Iowa, Wyoming, and Montana).

Wild waterfowl are a primary natural host for LPAIVs, and infection rates in these populations peak at autumn migratory staging locations, where large numbers of immunologically naive juvenile birds congregate (*4*). The HPAI H5N8 virus has apparently adapted to wild waterfowl hosts: few or no clinical signs or adverse effects are apparent in these hosts when infected with the virus. Thus, it seems probable that the virus was disseminated out of Russia into Europe, East Asia, and North America by migrating waterfowl during autumn 2014 (*5*).

The HPAI H5N8 virus has encountered, interacted with, and reassorted with co-circulating LPAIVs in migratory and overwintering waterfowl populations, creating new HPAI viruses (HPAIVs). In Taiwan, new Eurasian lineage reassortant HPAIVs (i.e., H5N2 and H5N3 subtypes) and the parental H5N8 subtype virus have been detected in poultry and wild birds (*6*). In North America, HPAI H5N8 virus continues to circulate among waterfowl and commercial and backyard poultry flocks. In addition, new HPAIV reassortants (i.e., H5N2 and H5N1 subtypes) that are combinations of HPAI H5N8 virus and genetic elements from Eurasian and North American viruses are also circulating in these populations (*7*,*8*) (Figure).

**Figure:**
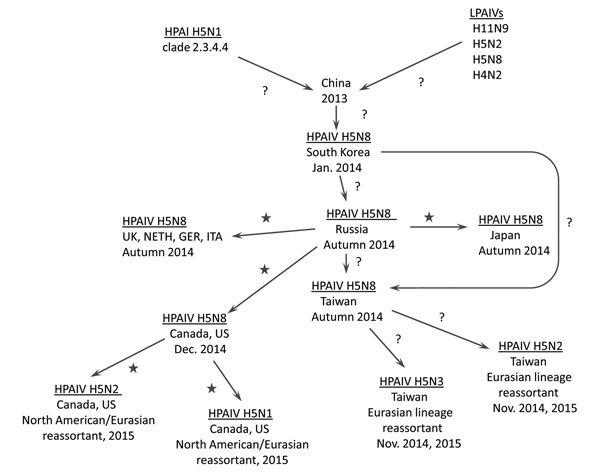
Genealogy of subtype H5N8 HPAIV, its spread from China to other countries, and its evolution in wild birds. Stars represent probable spread of virus and/or reassortment in wild birds; question marks indicate unknown mode. GER, Germany; HPAIV, highly pathogenic avian influenza virus; ITA, Italy; LPAIVs, low pathogenicity avian influenza viruses; Neth, the Netherlands; UK, United Kingdom; US, United States.

Persistence of the original HPAI H5N8 virus for >1 year, the creation of multiple reassortant viruses that have maintained high pathogenicity in poultry, and adaptation of the virus to migrating waterfowl all indicate that these viruses could persist and spread in Northern Hemisphere waterfowl populations for an extended period. This dynamic of HPAIVs being transported by wild birds to new populations raises critical issues and poses a series of questions that researchers and modelers should examine in more detail. The risks are significant that these HPAIVs will continue to circulate and that new genetic combinations will arise in concentrations of overwintering waterfowl and then spill over into poultry operations and aviculture. The spillover risk is particularly high for operations with rudimentary biosafety practices (e.g., backyard flocks) and that trend toward outdoor access for organic poultry. Such events have already occurred in commercial poultry operations in Canada and some US states (California, Minnesota, Missouri, Iowa, and Arkansas), and subsequent culling operations and trade restrictions have caused substantial local economic losses. As wild birds begin their spring migrations and disperse into their breeding ranges, will they be transporting these viruses to new regions, including the rest of North America? Is this an inevitable outcome of HPAI H5N8 transmission in wild bird populations? Can these viruses be transported from Europe to eastern North America by migratory birds via North Atlantic routes (*9*)? Are there risks of these viruses reassorting with viruses from other species, such as swine, particularly feral swine whose populations are rapidly expanding, and will these reassortant viruses present greater risk of zoonotic disease?

These HPAIVs do not appear to pose substantial risks to waterfowl populations, but they may have detrimental effects on other, perhaps more sensitive, wildlife populations. Birds of prey seem to be particularly susceptible to HPAIV infection (*10*), including the HPAIV H5N8 virus that killed captive gyrfalcons (*Falco rusticolus*) that were fed infected duck carcasses (*7*). In North America, other raptor species have been found infected with H5N8 or H5N2 virus: Cooper’s hawk (*Accipiter cooperii*), great horned owl (*Bubo virginianus*), red-tailed hawk (*Buteo jamaicensis*), peregrine falcon (*Falco peregrinus*), and bald eagle (*Haliaeetus leucocephalus*). It is not known what effect these viruses will have on small, at-risk wild bird populations, such as California condors (*Gymnogyps californianus*), that may prey on or scavenge infected birds, but the possible effects should be considered in conservation management decisions.

As HPAIVs continue spreading and evolving, the questions posed here, along with many more questions, will need to be answered to understand the risks to agriculture, zoologic collections, wildlife, and, potentially, human populations. As other researchers have recently pointed out, robust, targeted surveillance programs among wild birds (*11*) and poultry, modeling of the movements of HPAIV-infected wild birds, and experimental research studies will provide the knowledge required for intelligent policy and management decisions regarding agriculture, wildlife, and public health.
